# Mechanistic Insights of Neuroprotective Efficacy of Verapamil-Loaded Carbon Quantum Dots against LPS-Induced Neurotoxicity in Rats

**DOI:** 10.3390/ijms25147790

**Published:** 2024-07-16

**Authors:** Esraa M. Mosalam, Aya Ibrahim Elberri, Mahmoud S. Abdallah, Hend Mohamed Abdel-Bar, Abdel-Aziz A. Zidan, Hany A. Batakoushy, Hend E. Abo Mansour

**Affiliations:** 1 Biochemistry Department, Faculty of Pharmacy, Menoufia University, Shebin El-Kom 32511, Menoufia, Egypt; hend_elsaid@phrm.menofia.edu.eg; 2 Genetic Engineering and Molecular Biology Division, Department of Zoology, Faculty of Science, Menoufia University, Shebin El-Kom 32511, Menoufia, Egypt; ayaelbery62@science.menofia.edu.eg; 3 Clinical Pharmacy Department, Faculty of Pharmacy, University of Sadat City (USC), Sadat City 32897, Monufia, Egypt; 4 Department of Pharm D, Faculty of Pharmacy, Jadara University, Irbid 21110, Jordan; 5 Department of Pharmaceutics, Faculty of Pharmacy, University of Sadat City (USC), Sadat City 32897, Monufia, Egypt; hend.abdelbar@fop.usc.edu.eg; 6 Zoology Department, Faculty of Science, Damanhur University, Damanhur 22511, Beheira, Egypt; a.zidan@sci.dmu.edu.eg; 7 Department of Pharmaceutical Analytical Chemistry, Faculty of Pharmacy, Menoufia University, Shebin El-Kom 32511, Menoufia, Egypt; hany.batakoushy@phrm.menofia.edu.eg

**Keywords:** verapamil, CQDs, Alzheimer’s disease, LPS, CREB, CYP2B

## Abstract

Alzheimer’s disease (AD) is a neurodegenerative disease that badly impacts patients and their caregivers. AD is characterized by deposition of amyloid beta (Aβ) and phosphorylated tau protein (pTau) in the brain with underlying neuroinflammation. We aimed to develop a neuroprotective paradigm by loading verapamil (VRH) into hyaluronic acid-modified carbon quantum dots (CQDs) and comparing its effectiveness with the free form in an AD-like model in rats induced by lipopolysaccharide (LPS). The experimental rats were divided into seven groups: control, LPS, CQDs, early free VRH (FVRH), late FVRH, early verapamil carbon quantum dots (VCQDs), and late VCQDs. Characterizations of VCQDs, the behavioral performance of the rats, histopathological and immunohistochemical changes, some AD hallmarks, oxidative stress biomarkers, neuro-affecting genes, and DNA fragmentation were determined. VRH was successfully loaded into CQDs, which was confirmed by the measured parameters. VRH showed enhancement in cognitive functions, disruption to the architecture of the brain, decreased Aβ and pTau, increased antioxidant capacity, modifiable expression of genes, and a decline in DNA fragmentation. The loaded therapy was superior to the free drug. Moreover, the early intervention was better than the late, confirming the implication of the detected molecular targets in the development of AD. VRH showed multifaceted mechanisms in combating LPS-induced neurotoxicity through its anti-inflammatory and antioxidant properties, thereby mitigating the hallmarks of AD. Additionally, the synthesized nanosystem approach exhibited superior neuroprotection owing to the advantages offered by CQDs. However, finding new actionable biomarkers and molecular targets is of decisive importance to improve the outcomes for patients with AD.

## 1. Introduction

Lipopolysaccharide (LPS) is commonly utilized in animal studies to create AD-like models. LPS is a component of the outer membrane of Gram-negative bacteria and is known to trigger a severe inflammatory response in the central nervous system [1]. LPS works by activating microglial cells’ toll-like receptor 4 (TLR4), which causes the release and synthesis of pro-inflammatory cytokines, such as interleukin-6 (IL-6), tumor necrosis factor-alpha (TNF-α), and interleukin-1 beta (IL-1β) [2]. It is thought that LPS triggers an inflammatory response that aids in the emergence and advancement of AD pathology. Increased formation together with decreased clearance of Aβ and neuronal injury are possible outcomes of LPS-induced neuroinflammation and neurotoxicity [3].

A progressive neurodegenerative disease, Alzheimer’s disease (AD) is characterized by the build-up of neurofibrillary tangles and amyloid beta (Aβ) plaques in the brain, which impair neuronal functions and cause cognitive decline and eventually memory loss [4]. The prevalence of AD is rising worldwide; the issue presents a serious problem for healthcare systems everywhere and is of decisive importance. AD and dementia-related illnesses are the seventh most common causes of mortality and some of the main reasons why older people have disabilities [5]. Despite decades of research, the management of AD remains a significant challenge, as the available pharmacological interventions provide only modest and temporary symptomatic relief. Thus, there is a pressing need to investigate cutting-edge therapy approaches that can successfully halt or decrease the progression of AD.

The FDA has approved several drugs for the treatment of AD, primarily cholinesterase inhibitors, such as donepezil, rivastigmine, and galantamine, as well as the N-methyl-D-aspartate (NMDA) receptor antagonist memantine. These medications can temporarily improve or stabilize cognitive and functional abilities in patients with mild to moderate AD [6,7,8]. However, they do not halt or reverse the underlying disease progression, and their long-term efficacy is limited.

In recent years, there has been a growing focus on the development of novel therapeutic strategies for AD. These emerging approaches target various pathological hallmarks of the disease, including the accumulation of Aβ peptides and hyperphosphorylated tau proteins [8]. For instance, anti-amyloid agents aim to reduce Aβ production, promote Aβ clearance, or disrupt Aβ aggregation, while anti-tau therapies seek to stabilize or inhibit the hyperphosphorylation of tau, thereby preventing the formation of neurofibrillary tangles [9,10]. Additionally, neuroprotective agents that can mitigate oxidative stress, inflammation, and excitotoxicity have been investigated as potential treatments for AD [11,12]. Despite these promising developments, the translation of these novel therapeutic strategies into effective clinical interventions has been challenging. The multifaceted pathology of AD, involving complex interactions between various biochemical and neurological processes, underscores the need for continued research into innovative treatment approaches that can comprehensively address the underlying mechanisms of the disorder.

Nanotechnology has shown great promise in recent years for improving treatment efficacy, minimizing side effects, and delivering drugs in a tailored manner [13]. Some of these nanoformulations are semiconducting inorganic nanoparticles with distinct optical properties known as quantum dots (QDs). They have a number of benefits, including reduced toxicity, simplicity in formulation and processing, and extended stability in biological fluids. When they are designated for biological applications, stability, safety, and solubility become crucial considerations [14,15]. There are numerous varieties of QDs based on the core element, which can be cadmium, silver, indium, carbon, or silicon. A vital component of their nature and biosafety is the shell that surrounds the core element, which permits surface modification and bioconjugation [16].

Carbon quantum dots (CQDs) are a class of nanomaterials that have attracted a lot of interest because of their exceptional biocompatibility, low toxicity, and distinctive physicochemical characteristics. Fluorescent CQDs come in sizes smaller than 10 nm and are easily customized for the delivery and encapsulation of drugs [17,18]. The blood–brain barrier (BBB) can be easily crossed by CQDs, which enables targeted administration of therapeutics to the brain. Consequently, the effectiveness of medications can be increased and off-target effects can be decreased with this focused distribution [19]. CQDs have been effectively combined with various drugs, enhancing their therapeutic potential through improved delivery and efficacy. CQDs successfully improved the in vitro and in vivo efficacy of various chemotherapeutic agents, such as doxorubicin, methotrexate, 5-fluorouracil, paclitaxel, cisplatin, boldine, flutamide, and lisinopril [20,21,22].

Verapamil (VRH), a well-known calcium channel blocker, seemed to show potential neuroprotective effects in previous studies through different molecular mechanisms, such as reducing neurotoxicity caused by Aβ, restoring tau activity, and inhibiting calcium rise in microglial cells with further anti-inflammatory effects [23]. Moreover, because of its impact on P-glycoprotein, VRH can control the functions of the BBB and modify cellular calcium homeostasis, which may postpone the onset of AD [23]. Indeed, we previously investigated its ability to decrease hyperphosphorylated tau protein and lessen neuroinflammation with a modulatory effect on the PKA/CREB/BDNF signaling pathway [24]. Additionally, we examined the neuroprotection of VRH-loaded CQDs in vitro using SH-Sy5y and Neuro 2a neuroblastoma cell lines, and the results were sufficiently promising to extend the conceptualization to an in vivo experiment [25]. However, there exists an unmet need to discover more underlying molecular mechanisms that may be involved in VRH-inducing neuroprotection. In this context, we were the first to aim to examine the neuroprotective effect of VRH when loaded into CQDs in an animal model. Furthermore, we investigated the possible implicated pathways, which may be modulated by VRH to protect against LPS-induced neurotoxicity.

## 2. Results

### 2.1. Preparation of Hyaluronic Acid-Modified Carbon Quantum Dots

**Figure 1:**
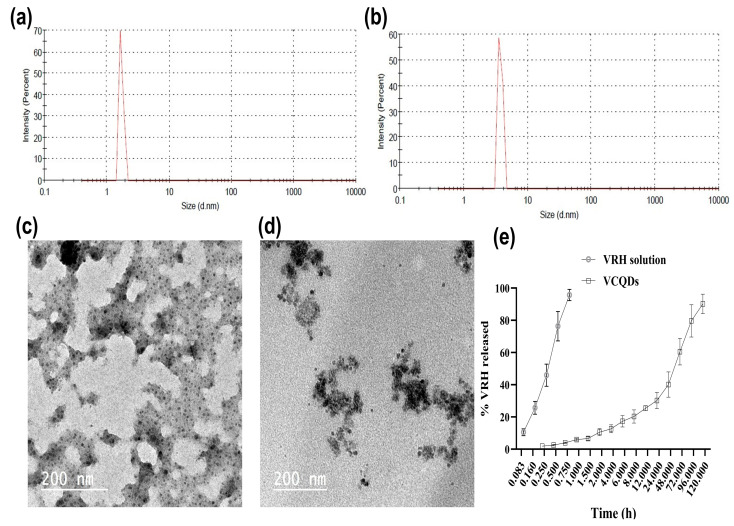
In vitro characterization of the prepared HA-CQDs and VCQDs. (**a**,**b**) Particle size histograms of HA-CQDs and VCQDs. Both HA-CQDs and VCQDs are monodisperse systems with particle sizes of 4.8 ± 0.23 nm and 7.5 ± 0.54 nm, respectively. (**c**,**d**) Transmission electron micrographs of HA-CQDs and VCQDs. Both systems appeared as spherical non-aggregated nanostructures with particle sizes consistent with the DLS measurements. (**e**) In vitro release of VRH from VCQDs and solution in PBS (pH 7.4) in the presence of FBS (50% *v*/*v*) at 37 °C. Drug release was measured by dialyzing HA-CQDs in the presence of 50% FBS against PBS (pH 7.4). Drug concentration in the dialysate was assessed by HPLC. Data points represent means and SDs (n = 3).

A single-step hydrothermal method was adopted to fabricate verapamil carbon quantum dots (VCQDs) using ascorbic acid as a precursor. Figure 1a,b show particle size histograms of the prepared HA-CQDs and VCQDs. The prepared HA-CQDs showed a particle size of 3.8 ± 0.45 nm. Table 1 shows the physicochemical properties of the VCQDs; a quantum yield % of the prepared VCQDs of 15.65 ± 2.54 was attained. The measured particle size was 7.5 ± 0.54 nm, which is in agreement with the reported particle size range of CQDs [26]. The measured polydispersity index (PDI) value of the fabricated VCQDs was 0.18 ± 0.012, indicating a unimodal particle size distribution [27]. The obtained VCQDs acquired a negative charge due to the presence of carboxylic groups on their surfaces [28]. Additionally, VRH could be successfully associated with CQDs with an association efficiency percentage (AE%) of 81.25 ± 3.65%. 

### 2.2. Transmission Electron Microscopy

The morphological architecture of the fabricated HA-CQDs and VCQDs appeared as scattered non-aggregated spherical nanostructures with particle sizes of 5–8 nm (Figure 1c,d). The measured sizes were in agreement with the data obtained from the DLS measurements.

### 2.3. In Vitro Drug Release

The in vitro release data presented in Figure 1e illustrate a stark contrast between the release profiles of the VRH solution and the fabricated VCQDs. The VRH solution exhibited a rapid release, achieving 95.69 ± 3.52% release within the first hour, indicative of an immediate release mechanism. In contrast, the VCQDs demonstrated a controlled and sustained release, with only 1.89 ± 0.23% release at 0.25 h, which gradually increased to 90.22 ± 5.98% over 120 h. This extended-release profile of the VCQDs suggests that HA-CQDs could provide a steady release over time. Such a sustained release is advantageous for maintaining therapeutic drug levels, potentially reducing dosing frequency and minimizing side effects [29].

### 2.4. Behavioral Tests

**Figure 2:**
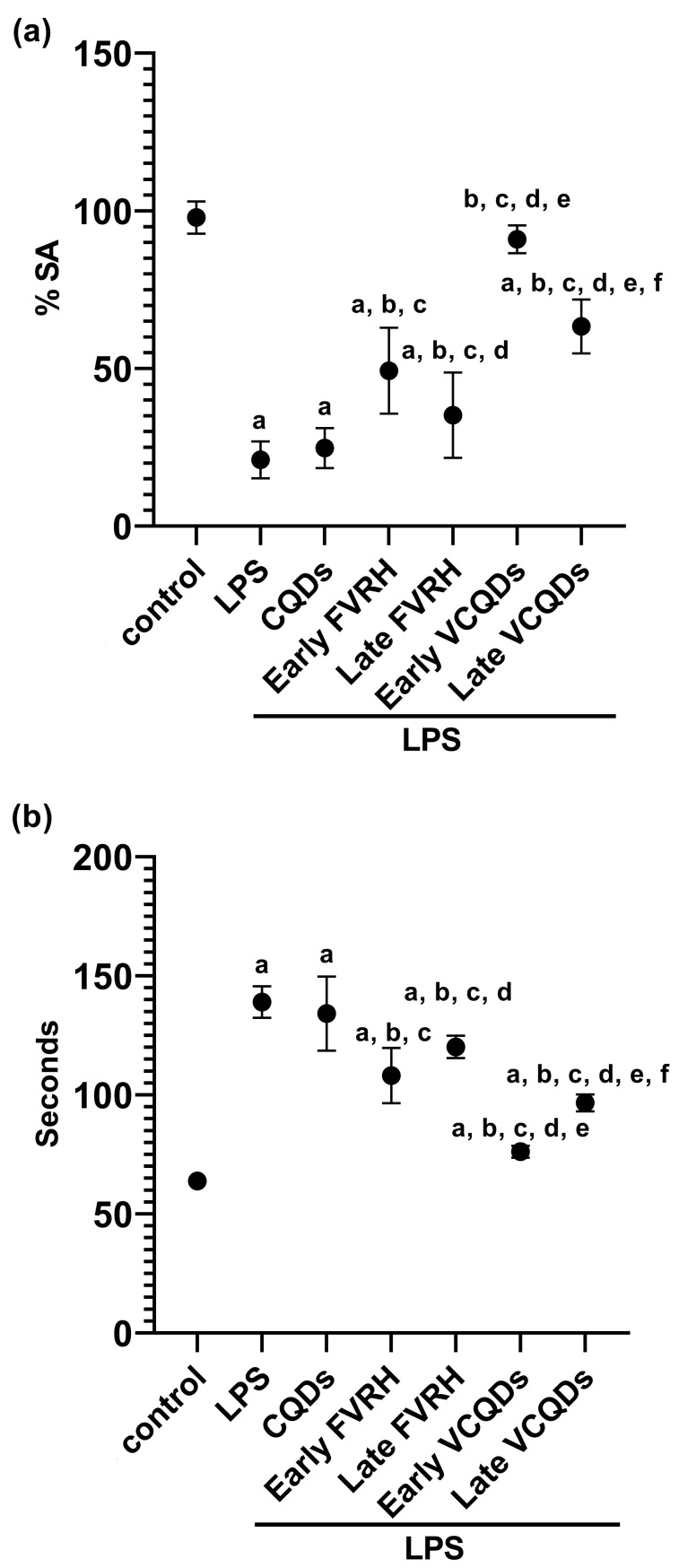
Effects on behavioral performance of the experimental rats: (**a**) Y-maze test and (**b**) pole-climbing test. Data are presented as means, n = 6, *p* < 0.05. a: significant vs. control, b: significant vs. LPS, c: significant vs. CQDs, d: significant vs. early FVRH, e: significant vs. late FVRH, f: significant vs. early VCQDs. LPS: lipopolysaccharide, CQDs: hyaluronic acid caron quantum dots, FVRH: free verapamil, VCQDs: verapamil-loaded CQDs, %SA: percentage of spontaneous alteration.

LPS negatively impacted the behavioral performance of the rats, as presented in Figure 2. In the Y-maze test, there was a significant (*p* < 0.001) decrease in %SA and a significant (*p* < 0.001) increase in time in the pole-climbing test compared to the control. Protection of the rats by the free VRH reversed these findings in the early (*p* < 0.001) and late phases (*p* = 0.004) of the protection. Similarly, the loaded VRH significantly (*p* < 0.001) amended these disabilities whether it was administered early or late compared to the LPS group. The loaded VRH therapies showed significant results (*p* < 0.001) compared to their corresponding free forms.

### 2.5. Histopathological Examination

**Figure 3:**
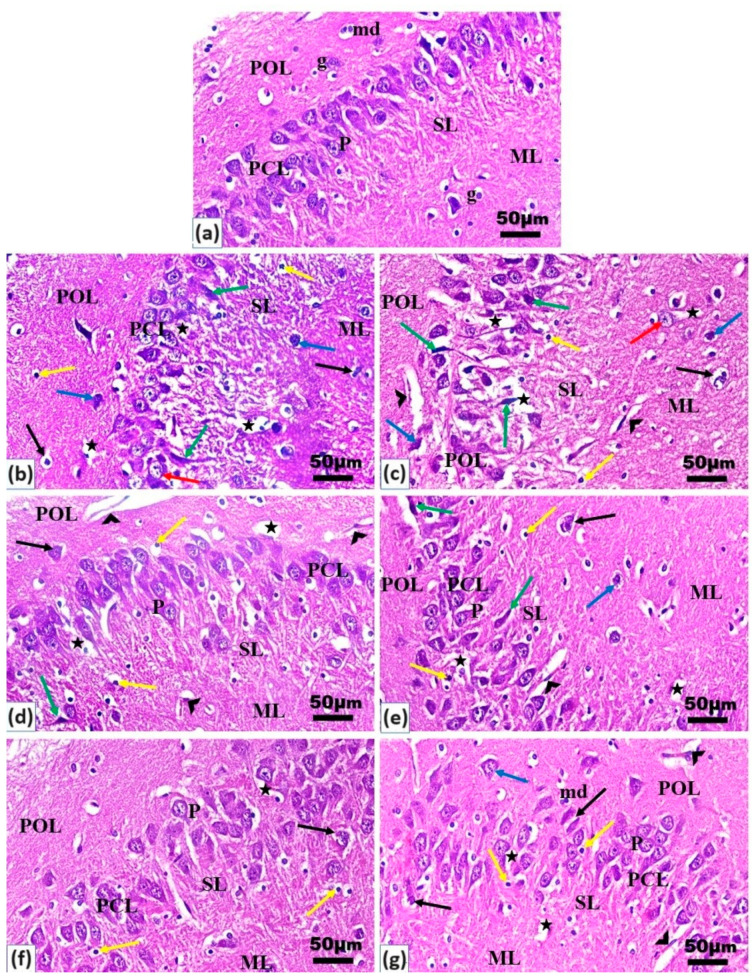
Histological microphotographs of the brains of rats stained with hematoxylin and eosin (H&E): (**a**) control, (**b**) LPS, (**c**) CQDs, (**d**) early FVRH, (**e**) late FVRH, (**f**) early VCQDs, and (**g**) late VCQDs. Groups represented by (**c**–**g**) received LPS. Groups presented different hippocampus, polymorphic (POL), pyramidal cell (PCL), septum lucidum (SL), and molecular (ML) layers. P: pyramidal neuron, g: glial cell, md: mitotic division; arrowheads: blood capillaries, green arrows: perineural oligodendrocytes with deep-stained elongated/flat nuclei, red arrows: large light-stained nuclei, black arrows: apoptotic cells, yellow arrows: pyknotic nuclei, blue arrows: karyorrhexic nuclei, stars: intercellular and pericellular vacuolation (H&E, ×400). LPS: lipopolysaccharide, CQDs: hyaluronic acid caron quantum dots, FVRH: free verapamil, VCQDs: verapamil-loaded CQDs.

Brain sections of H&E-stained slices of the control rats revealed normal layers: the polymorphic layer contained deep-stained glial neurons with mitotic division, the pyramidal cell layer contained pyramidal neurons, the septum lucidum layer contained deep and light-stained nuclei of glial cells, and the molecular layer contained light-stained glial neurons (Figure 3a). The LPS group exhibited several histopathological abnormalities and degeneration in all the layers, especially the pyramidal cell and septum lucidum layers, such as dilation of the blood capillaries, intercellular and pericellular vacuolation, perineural oligodendrocytes with deep-stained elongated/flat nuclei, large cells with light-stained nuclei, apoptotic cells with degenerated nuclei, and necrotic cells with either dark condensed pyknotic nuclei or karyorrhexic (fragmented) nuclei (Figure 3b). Rats from all the pretreated groups—the CQD, early FVRH, late FVRH, early VCQD, and late VCQD groups—displayed improvement when compared with the previous alterations in the LPS group (Figure 3c–g). The CQD, late FVRH, early VRH, late VCQD, and early VCQD groups exhibited severe, high, moderate, mild, low, rare, or absent histopathological abnormalities, respectively, as presented in Figure 3 and Table 2.

### 2.6. Immunohistochemistry Examination

**Figure 4:**
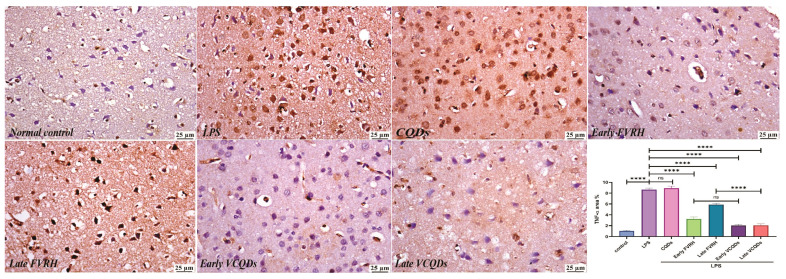
Histological microphotographs of the brains of rats immunostained with anti-TNF-α (×400). Data are presented as means ± SE, n = 5, *p* < 0.05, **** (*p* < 0.0001), ns: non-significant. TNF-α: tumor necrosis factor-alpha, LPS: lipopolysaccharide, CQDs: hyaluronic acid caron quantum dots, FVRH: free verapamil, VCQDs: verapamil-loaded CQDs.

**Figure 5:**
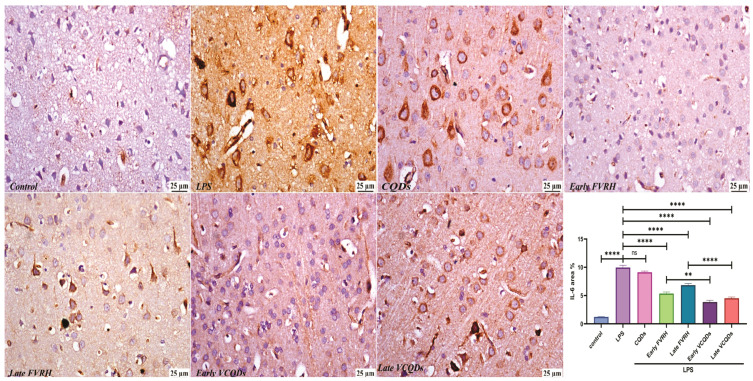
Histological microphotographs of the brains of rats immunostained with anti-IL-6 (×400). Data are presented as means ± SE, n = 5, *p* < 0.05, **** (*p* < 0.0001), ** (*p* < 0.01), ns: non-significant. IL-6: interleukin 6, LPS: lipopolysaccharide, CQDs: hyaluronic acid caron quantum dots, FVRH: free verapamil, VCQDs: verapamil-loaded CQDs.

**Figure 6:**
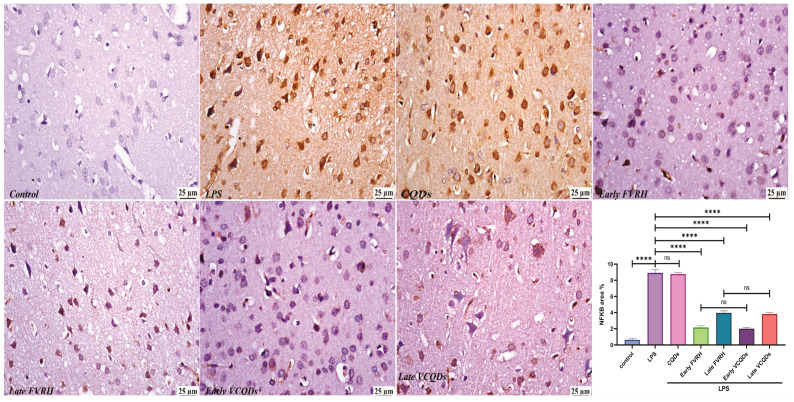
Histological microphotographs of the brains of rats immunostained with anti-NF-κB (×400). Data are presented as means ± SE, n = 5, *p* < 0.05, **** (*p* < 0.0001), ns: non-significant. NF-κB: nuclear factor kappa-light-chain-enhancer of activated B cells, LPS: lipopolysaccharide, CQDs: hyaluronic acid caron quantum dots, FVRH: free verapamil, VCQDs: verapamil-loaded CQDs.

**Figure 7:**
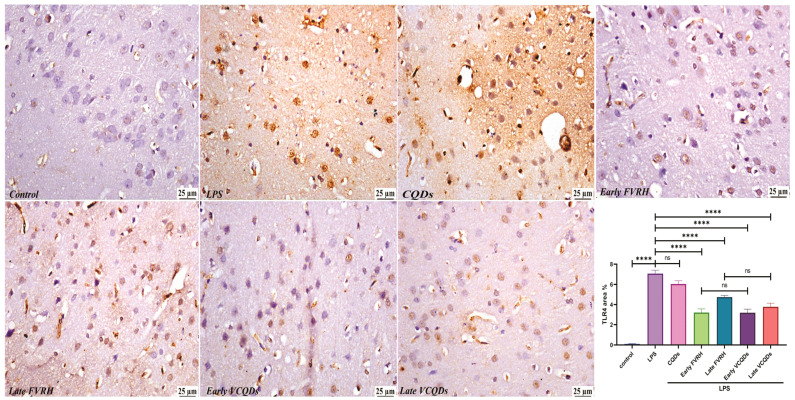
Histological microphotographs of the brains of rats immunostained with anti-TLR4 (×400). Data are presented as means ± SE, n = 5, *p* < 0.05, **** (*p* < 0.0001), ns: non-significant. TLR4: toll like receptor 4, LPS: lipopolysaccharide, CQDs: hyaluronic acid caron quantum dots, FVRH: free verapamil, VCQDs: verapamil-loaded CQDs.

**Figure 8:**
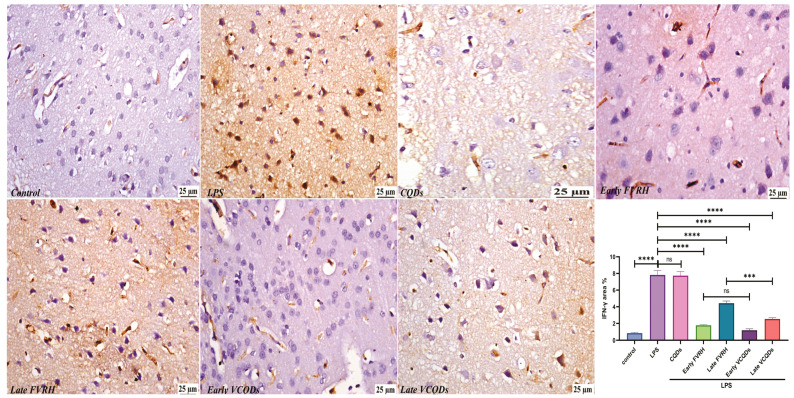
Histological microphotographs of the brains of rats immunostained with anti-IFN-γ (×400). Data are presented as means ± SE, n = 5, *p* < 0.05, **** (*p* < 0.0001), *** (*p* < 0.001), ns: non-significant. IFN-γ: interferon gamma, LPS: lipopolysaccharide, CQDs: hyaluronic acid caron quantum dots, FVRH: free verapamil, VCQDs: verapamil-loaded CQDs.

**Figure 9:**
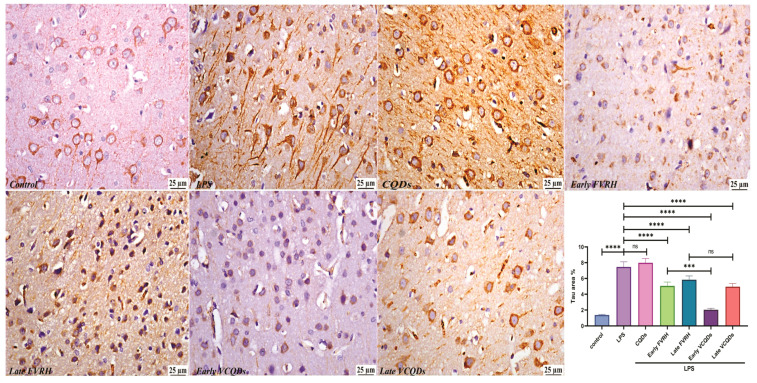
Histological microphotographs of the brains of rats immunostained with anti-pTau (×400). Data are presented as means ± SE, n = 5, *p* < 0.05, **** (*p* < 0.0001), *** (*p* < 0.001), ns: non-significant. pTau: phosphorylate-tau protein, LPS: lipopolysaccharide, CQDs: hyaluronic acid caron quantum dots, FVRH: free verapamil, VCQDs: verapamil-loaded CQDs.

There was a significant (*p* < 0.001) elevation in the levels of TNF-α (Figure 4), IL-6 (Figure 5), NF-κB (Figure 6), TLR4 (Figure 7), IFN-γ (Figure 8), and pTau (Figure 9) in the LPS group compared to the control. Pretreatment of the rats by VRH in the free or loaded form early or late decreased the levels of these biomarkers compared to the LPS group. The greatest reduction was observed in early VCQDs, which was significant (*p* < 0.05) against early free VRH in terms of IL-6 and pTau. The late VRH also exhibited significant (*p* < 0.05) results compared to the corresponding free form regarding TNF-α, IL-6, and IFN-γ.

### 2.7. Effect on Oxidative Stress Biomarkers

**Figure 10:**
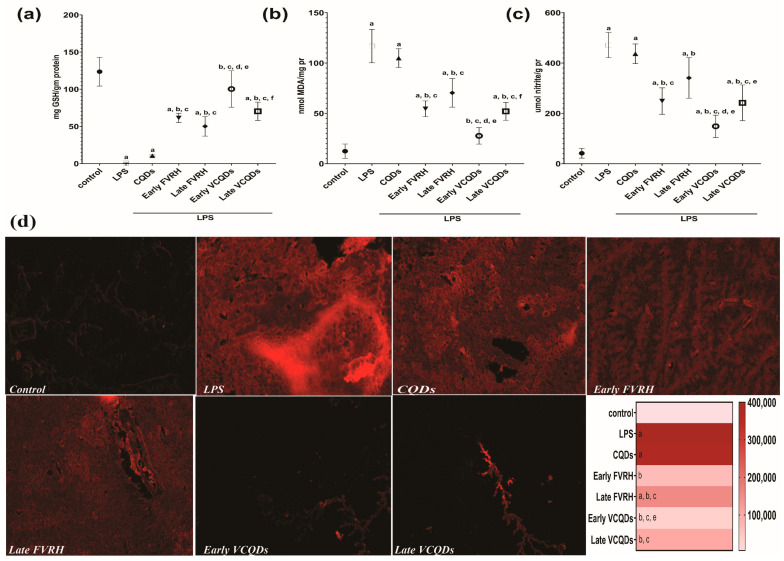
Effects on oxidative stress biomarkers: (**a**) concentration of GSH, (**b**) concentration of MDA, (**c**) concentration of nitrite, and (**d**) ROS fluorescence signals (bright red) and heatmap presentation for CTCF. Data are presented as means, n = 6, *p* < 0.05. a: significant vs. control, b: significant vs. LPS, c: significant vs. CQDs, d: significant vs. early FVRH, e: significant vs. late FVRH, f: significant vs. early VCQDs. LPS: lipopolysaccharide, CQDs: hyaluronic acid caron quantum dots, FVRH: free verapamil, VCQDs: verapamil-loaded CQDs, GSH: glutathione, MDA: malondialdehyde, ROS: reactive oxygen species.

Induction of neuroinflammation by LPS showed a significant (*p* < 0.001) decrease in the concentration of GSH with a significant (*p* < 0.001) elevation in the levels of MDA, nitrite, and ROS compared to the control. Protection of the rats by the early and late free VRH showed a significant (*p* < 0.001) increase in the level of GSH together with a significant (*p* < 0.001) decrease in MDA, nitrite, and ROS compared to the LPS group, but the early intervention was superior, with no significant difference between the two groups. Similarly, the early protection by VCQDs significantly (*p* < 0.001) elevated the GSH with a significant (*p* < 0.001) reduction in the levels of MDA, nitrite, and ROS compared to LPS. The same effect was observed in the late VCQD group, but there was a significant difference between this group and the corresponding early one in terms of GSH (*p* = 0.010) and MDA (*p* = 0.007), though not for nitrite (*p* = 0.072) and ROS (*p* = 0.290), as shown in Figure 10.

It is worth noting that the early VCQDs revealed significant results against the early free drug for GSH (*p* < 0.001), MDA (*p* = 0.002), and nitrite (*p* < 0.042) but not for ROS. Comparing the two late therapies, significant results were only observed for the levels of nitrite (*p* = 0.046). There was also a noticeable rise in the level of GSH together with a reduction in the levels of MDA, nitrite, and ROS in the group pretreated with CQDs relative to the LPS group, as presented in Figure 10.

### 2.8. Effect on Amyloid Beta

**Figure 11:**
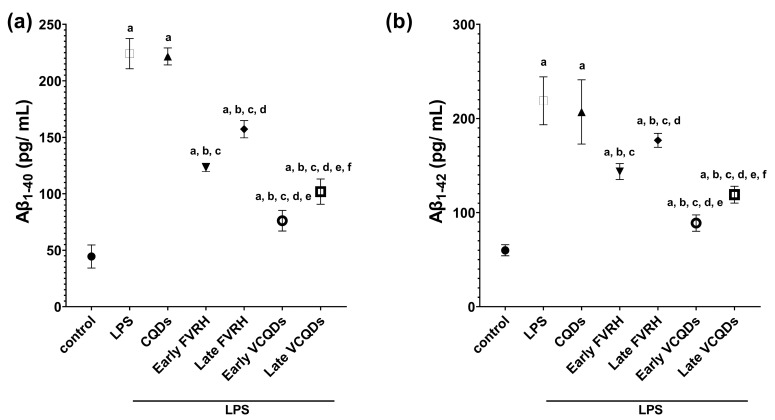
Effects on amyloid beta (Aβ) proteins: (**a**) concentration of Aβ_1–40_ and (**b**) concentration of Aβ_1–42_. Data are presented as means, n = 6, *p* < 0.05. a: significant vs. control, b: significant vs. LPS, c: significant vs. CQDs, d: significant vs. early FVRH, e: significant vs. late FVRH, f: significant vs. early VCQDs. LPS: lipopolysaccharide, CQDs: hyaluronic acid caron quantum dots, FVRH: free verapamil, VCQDs: verapamil-loaded CQDs.

Figure 11 shows that the concentrations of the two detected sequences of Aβ protein were significantly (*p* < 0.001) elevated in the LPS group relative to the control. Protection of the rats by the free or loaded VRH early or late showed a significant (*p* < 0.001) reduction in the levels of the detected Aβ proteins. There was also a significant difference between the early and late protected rats (*p* = 0.002 and *p* = 0.005, respectively, for the free and the loaded drug); the effect of the early intervention was more pronounced than that of the late one. Comparing the loaded therapy with the free form, there was a statistically significant difference (*p* < 0.001) regarding both sequences of Aβ protein. In an unexpected manner, there was a more inhibitory effect on Aβ_1–40_ than on Aβ_1–42_.

### 2.9. qRT-PCR for Genes’ Expression

**Figure 12:**
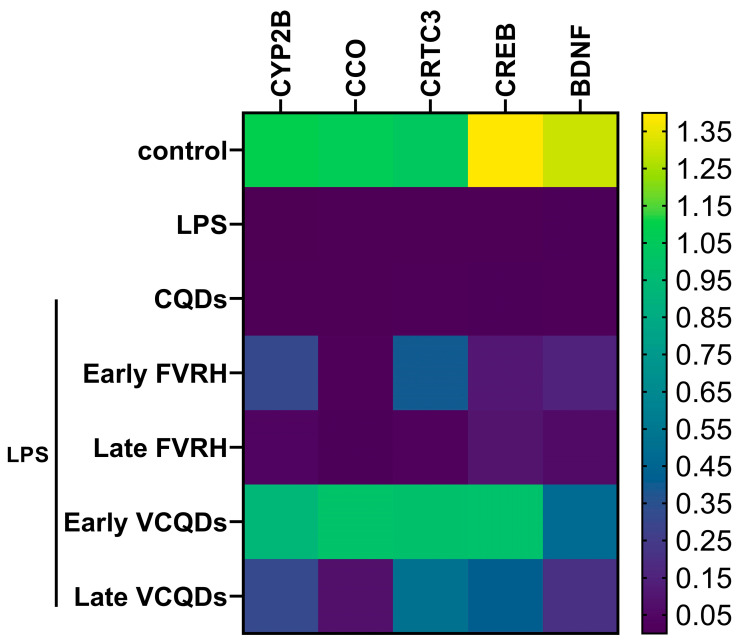
Heatmap presentation of the relative quantification (RQ) of the detected genes. LPS: lipopolysaccharide, CQDs: hyaluronic acid caron quantum dots, FVRH: free verapamil, VCQDs: verapamil-loaded CQDs, CYP1A1: cytochrome P1A1, CCO: cytochrome c oxidase, CRTC3: CREB-regulated transcriptional coactivator 3, CREB: cAMP response element-binding protein, BDNF: brain-derived neurotrophic factor.

Induction of neuroinflammation by LPS showed a significant (*p* < 0.0001) downregulation of CYP2B, CCO, CRTC3, CREB, and BDNF compared to the control group. Protection of the rats by the early and late free or loaded VRH showed significant upregulation for CREB (*p* = 0.027, *p* = 0.029, *p* < 0.0001, and *p* = 0.001, respectively, for the groups). Similarly, the pretreatment of the rats with the early free VRH, the early VCQDs, and even the late VCQDs revealed significant upregulation of the expression of CYP450 (*p* = 0.002, *p* < 0.001, and *p* = 0.003, respectively, for the groups), CRTC3 (*p* = 0.002, *p* < 0.001, and *p* = 0.002, respectively, for the groups), and BDNF (*p* = 0.01, *p* < 0.001, and *p* = 0.002, respectively, for the groups). Concerning the expression pattern of CCO, there was a significant upregulation only in the groups pretreated with early VCQDs (*p* < 0.0001) and late VCQDs (*p* = 0.002) compared to the LPS group. There was a significant difference in the expression of CCO between the free and loaded therapies in the early (*p* = 0.048) and the late (*p* = 0.043) phase of the protection. The same effect was observed in the expression of CREB but only in the early stage of the protection (*p* = 0.048). There was also superior upregulation in the expression levels of these genes by the early pretreatment relative to the late ones, but the difference was non-significant. Table S2 shows the medians and interquartile ranges of the detected genes for the studied groups, and Figure 12 shows a heatmap of the expression patterns of the genes.

### 2.10. Effect on DNA Fragmentation

**Figure 13:**
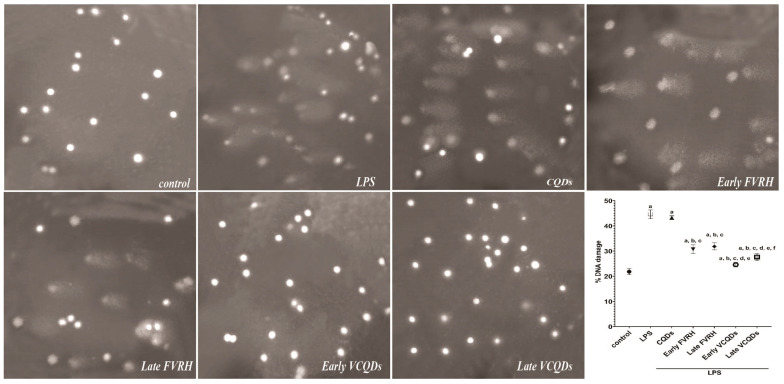
Effects on DNA fragmentation detected by comet assay (×400). Data are presented as means, n = 6, *p* < 0.05. a: significant vs. control, b: significant vs. LPS, c: significant vs. CQDs, d: significant vs. early FVRH, e: significant vs. late FVRH, f: significant vs. early VCQDs. LPS: lipopolysaccharide, CQDs: hyaluronic acid caron quantum dots, FVRH: free verapamil, VCQDs: verapamil-loaded CQDs.

Figure 13 shows that the percentage of DNA fragmentation was significantly increased by LPS (*p* < 0.0001) compared to the control group. Protection of the rats early or late by the free and loaded VRH significantly (*p* < 0.0001) decreased the level of fragmentation relative to the LPS group. There was a significant (*p* < 0.0001) decrease in the damage observed in the groups pretreated with VCQDs early or late compared to those pretreated with the free corresponding drug. There was also a significant (*p* = 0.007) reduction in the DNA damage in the early VCQD-pretreated group compared to the late VCQD-pretreated one.

## 3. Discussion

To the best of our knowledge, this is the first study to have examined the neuroprotective effect of VRH-loaded CQDs in a rat model of neuroinflammation induced by LPS. Moreover, we aimed to elucidate the incorporation of multiple molecular targets in the development of neuroinflammation, which could be a crucial mechanism in neurodegenerative diseases such as AD.

The role of neuroinflammation in neurodegenerative diseases was initially considered by McGeer et al. (1988), who revealed that activated microglial cells are responsible for the neuroinflammatory cascade [30]. LPS primarily induces neuroinflammation through binding to TLR4, a microglia receptor. The activated TLR4 in turn activates the NF-κB and activator protein-1 (AP-1) signaling pathways, which leads to the release of inflammatory cytokines, such as TNF-α, IL-6, IL-1β, and IFN-γ [31,32]. The activation of microglial cells was also found to be linked to hyperphosphorylation of tau protein and decreased clearance of amyloid plaques [32]. In this context, the LPS group in our study exhibited perturbations in the histological morphology of the brain, increased precipitated Aβ, and positive reactivity toward the antibodies of TNF-α, IL-6, NF-κB, TLR4, IFN-γ, and pTau. Our results are in harmony with other studies which demonstrated the neuropathy of LPS through binding to TLR4 [3,31,33], activation of NF-κB [31,34] followed by the release of inflammatory cytokines [24,33,34,35], and ultimately taupathy [24,36,37,38] and amyloid plaque deposition [3,33,36]. Protection of the rats by VRH alleviated the inflammatory environment, which could be attributed to the anti-inflammatory effect of VRH that is mediated through suppression of activated microglial cells, thereby blocking the release of pro-inflammatory cytokines [24]. VRH can also downregulate TLR4 together with inhibition of the NF-κB signaling pathway [39]. Our findings are in line with preceding research which indicated the anti-inflammatory effect of VRH using in vitro and in vivo models [40,41,42,43].

The brain is vulnerable to oxidative stress owing to the limited capacity of the antioxidant defense system and constrained regeneration. Moreover, there is crosstalk between neuroinflammation and oxidative stress, which can lead to further damage. Indeed, activated microglia produce proinflammatory cytokines, oxidative enzymes, and ROS as a defense in the beginning, but later this become overzealous and neurotoxic [44]. This can explain the increased levels of MDA, nitrite, and ROS and depletion of GSH, which reflect that the antioxidant capacity was overwhelmed by LPS. Our findings are in line with previous studies which indicated that LPS induces oxidative damage [33,35,45,46]. In contrast, the VRH-pretreated rats revealed diminished levels of oxidative stress biomarkers and increased levels of GSH. This favorable neuroprotection of VRH is mediated by its antioxidant property, which could be ascribed to the inhibitory action on microglia. Moreover, it is believed that there is a relationship between imbalanced calcium homeostasis, which could be induced by LPS, and the generation of free radicals and lipid peroxidation [24]. Therefore, restoring the calcium pool by VRH can result in reinstatement of the antioxidant capacity of cells. These results are consistent with others that referred to the antioxidant effect of VRH [39,40,47,48].

The expression patterns of CYPs in the brain vary, and some CYPs are expressed in the brain more than in other sites. For example, CYP2B is principally expressed in the neuronal cells of the cerebellum, hippocampus, and thalamus, and it is responsible for metabolism of steroidal agents and other drugs. The CYP2B subfamily is under regulation by constitutive androstane receptors (CARs) and pregnane X receptors (PXRs) [49]. The downregulatory effect of LPS on the expression of several CYPs is believed to be related to the induced inflammation and suppression of CARs and PXRs [50,51]. Our findings are consistent with other outcomes that revealed the inhibitory effect of LPS on CYP2B [50,52], CYP1A [51,53,54], and CYP3A4 [51]. Pretreatment with VRH upregulated the expression of CYP2B, which is consistent with another study that indicated the stimulatory effect of some calcium channel blockers, including VRH, on some CYPs [55]. Moreover, H. Rosenbrock et al. stated that these CYPs play a protective role in neuronal cells against cytotoxic compounds such as LPS [56]. Concerning CCO, it is a mitochondrial respiratory enzyme (complex IV), and it has been found that CCO deactivation is a principal brain mitochondrial deficit in patients with AD as a result of accumulated amyloid plaques [57]. In alignment with our results, LPS showed an inhibitory effect on CCO by increasing the level of inducible nitric oxide synthase (iNOS) [58]. The VRH-pretreated groups revealed restoration of CCO expression. This beneficial effect could be attributed to the reinstatement of mitochondrial respiration through the balancing of calcium homeostasis by VRH, as suggested by our previously published data [24]. In addition, VRH has been found to inhibit iNOS due to inhibition of NF-κB [39].

Additionally, CREB-regulated transcription coactivator 1 (CRTC1) is a co-transcription factor that is primarily expressed in the brain. CRTC1 improves the binding of CREB to the gene promoter, thereby activating downstream target pro-survival proteins, including BDNF [59]. Consistent with our findings, S. Ni et al. also reported the repressive effect of LPS on CRTC1, which resulted in depressive behavior in mice [60]. As a result of inhibition of CRTC3, the activity of CREB and BDNF was dampened. We previously demonstrated the repressive effect of LPS on CREB and BDNF as a consequence of downregulation of protein kinase A (PKA) [24]. Additionally, there was reduced expression of BDNF in research that used CRTC1 knockout mice [61]. Other experiments also made the same finding regarding the effect of LPS on CREB and BDNF [62,63]. On the other hand, it is well known that imbalanced calcium homeostasis is highly linked to mitochondrial dysfunction, impaired synaptic plasticity, and calcium-induced neuronal apoptosis. Consequently, blocking of calcium influx by VRH could reverse these unfavorable events [41]. Increased synaptic plasticity and activity could activate CRTC1 with further activation of CREB/BDNF signaling [64]. This activation of CREB/BDNF by VRH was previously established by S. Ponne et al. [47]. Despite these explanations of the effect of VRH on the CRTC/CREB/BDNF signaling pathway, the exact mechanism has remained elusive and requires much more investigation.

Regarding the effect of LPS on DNA fragmentation, it was suggested that the unpropitious effect is mediated by increased formation of NO via iNOS, which in turn reacts with superoxide radicals and generates ROS. This oxidative insult leads to increased DNA damage and fragmentation [65]. Our results are also in alignment with another study which indicated increased chromatin condensation and DNA fragmentation by LPS in astrocytes [46]. All of the mentioned perturbations caused by the neurotoxic effect of LPS were reflected by modification of the behavior of the experimental rats by decreasing % SA as well as increasing the time in pole-climbing tests. Our findings are in line with previously published results about the modulatory effect of LPS in different behavioral tests [24,31,38]. On the other hand, oxidative stress has been linked to the triggering of calcium channels and increased calcium levels [66]. Consequently, blocking of calcium channels by VRH could reverse this adverse event and mitigate the DNA damage caused by LPS. A study carried out by V.M.M. Achary et al. supports our findings regarding the positive modulatory effect of VRH on DNA fragmentation [67]. The observed neuroprotective effect of VRH was also obvious in the behavioral tests, and these outcomes were confirmed by several previous studies which revealed enhanced behavioral performance of experimental rodents [24,41,47,68].

Our results demonstrated that the loaded VRH showed superior activity in the measured parameters and in the behavioral tests compared to the free drug. This beneficial effect could be ascribed to the CQDs. These carbon-based nanoformulations have gained great attention recently due to their simple preparation, good stability, small size, exceptional optical characteristics, high biocompatibility, good cell membrane permeability, low cytotoxicity, and functionalization capability [69,70]. Furthermore, CQDs can serve as excellent drug delivery systems in the brain due to their ability to cross the blood–brain barrier (BBB). Indeed, CQDs can cross the BBB without any functionalization [70,71], though in our study the CQDs were conjugated with hyaluronic acid (HA-CQDs), which could offer additional effective compatibility with brain tissue [72]. The same results were obtained by preceding researchers who used different forms of CQDs and proved their effectiveness in lowering precipitated pTau and Aβ [69,73,74]. It is worth mentioning that the CQD group showed a notable decrease in the levels of inflammatory mediators and detected ROS. Our previous findings also revealed that VRH-loaded CQDs may act as a promising candidate for neuroprotection against Aβ-induced neurotoxicity in SH-Sy5y and Neuro 2a neuroblastoma cell lines with great antioxidant and anti-inflammatory activities [25]. These outcomes are in line with others reported on the anti-inflammatory [75] and ROS-scavenging properties of CQDs [76,77]. Regarding the different time-treated groups, the early intervention was better at restoring the deteriorated biomarkers, and this effect was more obvious in the CQD therapies compared to the control or LPS groups. These findings confirm that the investigated molecular pathways are highly associated with AD development and progression and may represent promising targets in AD. Nevertheless, more investigations are attempting to decipher the obscure mechanisms of AD pathophysiology, establishing novel strategies to surmount this disease and improve outcomes for patients grappling with AD.

## 4. Materials and Methods

### 4.1. Materials

Hyaluronic acid sodium salt from Streptococcus equi (HA), ascorbic acid, methanol (HPLC grade), acetonitrile (HPLC grade), fetal bovine serum (FBS), and phosphate-buffered saline (PBS), and LPS were purchased from Merck (Kenilworth, Warwickshire, UK). Verapamil hydrochloride (>99%, VRH) was obtained from Abcam (Waltham, MA, USA). KH_2_PO_4_ and H_3_PO_4_ were purchased from Fluka Chemika BioChemika (Buchs, Switzerland). NaCl, Na_2_EDTA, low-melting-point agarose (LMA), and ethidium bromide (EP) were purchased from Avantor Performance Materials Poland S.A. (Gliwice, Poland).

### 4.2. Preparation and Characterization of Hyaluronic Acid-Modified Carbon Quantum Dots

#### 4.2.1. Preparation of Verapamil-Loaded Hyaluronic Acid-Modified Carbon Quantum Dots 

Verapamil-loaded hyaluronic acid-modified carbon quantum dots (VCQDs) were fabricated using a hydrothermal technique [78]. Briefly, HA (1% *w*/*v*) and ascorbic acid (4% *w*/*v*) were dissolved in deionized water by stirring for 1 h at 500 rpm. Subsequently, the obtained solution (10 mL) was poured into a 25 mL stainless-steel autoclave vessel. The vessel was heated in a furnace to 210 °C for 2 h. Afterwards, the dispersion was allowed to cool down to room temperature. The obtained HA-CQDs were purified by ultrafiltration centrifugation using Amicon^®^ Ultra filters (3500 Da) at 12,000 rpm for 15 min, followed by dialysis against deionized water (MWCO, 1000 Da) for 24 h [78]. Afterwards, VCQDs were prepared by mixing the collected HA-CQDs (40 mg/mL) with aqueous VRH (20 mg/mL) in a thermostatically controlled shaking water bath (Daihan Labtech Shaker Water Bath, LSB 030S, Namyangju, Gyeonggi, Republic of Korea) at 250 strokes ± 0.1 at 25 ± 0.5 °C for 24 h. The free unadsorbed VRH was removed by dialysis (MWCO, 1000 Da) against deionized water for 1 h.

#### 4.2.2. Determination of Quantum Yield 

The fluorescence spectra of serial concentrations of VCQDs in deionized water (0.5–2.5 µg/mL) and quinine sulfate in 0.1 M sulfuric acid as a standard were recorded at a λ excitation of 340 nm, and the integrated photoluminescence intensities (excited at 340 nm) were plotted against the absorbance values at 340 nm [79]. Quantum yield was calculated using the following equation:

Φx = ΦST (mx/mST) (η2x/η2ST)(1)

where Φ is the quantum yield, ΦST = 0.54, m is the slope, η is the refractive index of the solvent, ST is the standard, and X is the sample.

#### 4.2.3. Determination of Particle Size and Zeta Potential

The particle sizes and polydispersity indices (PDIs) of the VCQDs were measured by dynamic light scattering (DLS) using a particle size analyzer (Zetasizer Nano ZS, Malvern Instruments Ltd., Malvern, Worcestershire, UK). Zeta potential was quantified using electrophoresis. All the measurements represent the average of 20 runs; each run was completed in triplicate at 25° [80].

#### 4.2.4. Determination of Verapamil Association Efficiency 

The association efficiency percentage (AE%) of VRH was quantified by dissolving the prepared VCQDs in methanol. Subsequently, the amount of associated VRH was measured using a previously validated HPLC method [81]. The AE% was calculated using the following equation:

(2) $$\mathrm{AE}\%=\frac{amount\;of\;VRH\;inside\;the\;HA-CQDsVER}{Total\;amount\;of\;VER\;added}\times 100$$

#### 4.2.5. Transmission Electron Microscopy

The morphological architecture of the prepared VCQDs was visualized using a transmission electron microscope (TEM; Jeol, JEM-1230, Tachikawa, Tokyo, Japan) after staining with one drop of 1% phosphotungstic acid at 200 KV [82].

#### 4.2.6. In Vitro Drug Release

VRH release from the prepared VCQDs was assessed using the dialysis membrane method [78]. An aliquot of VCQDs (equivalent to 5 mg VRH) was suspended in a pre-soaked dialysis membrane (cutoff: 1000 Da) and mixed with 1 mL of PBS (pH 7.4) containing FBS (50% *v*/*v*). The tightly closed membranes were immersed in glass containers filled with 50 mL PBS (pH 7.4) and incubated in a thermostatically controlled shaking water bath set at 250 ± 0.1 strokes/min and 37 ± 0.5 °C. For comparison, the drug release assessment of the free VRH solution (5 mg/mL) was performed under the same conditions. At predetermined intervals, 0.5 mL aliquots were collected and replaced with medium. The VRH concentration was analyzed using HPLC as previously described, and the percentage of VRH released was estimated [81].

### 4.3. In Vivo Experiment

#### 4.3.1. Experimental Rats and Groupings

A total of forty-two male albino rats, aged 8–10 months and weighing 200–300 g, were obtained from the Egyptian Organization for Biological Products and Vaccines (VACSERA, Cairo, Egypt). The rats underwent a one-month adaptation period, during which they were provided with tap water, a veterinary diet, and day–night cycles. The housing and handling of the animals followed the guidelines outlined in the “Guide for the Care and Use of Laboratory Animals” produced by the National Research Council. The research proposal was approved by the Research Ethical Committee of the Faculty of Pharmacy, Menoufia University, Egypt, with the approval number MPIR241.

Randomly, the rats were divided into seven groups, each with six rats. The groups were as follows: control, LPS, CQDs, early free VRH (FVRH), late FVRH, early VCQDs, and late VCQDs. An AD-like neuroinflammatory condition was induced by injecting LPS dissolved in normal saline at a dosage of 750 μg/kg, intraperitoneally, on seven consecutive days [24]. Regarding the CQD group, each rat received 1 mL of CQDs for 7 days. The FVRH groups received intraperitoneal injections of VRH at a dosage of 10 mg/kg [24], either 4 days before the induction (early FVRH) or concomitantly with it (late FVRH). Similarly, the VCQD groups received 1 mL of VCQDs containing 3 mg of the free drug at the same time points.

#### 4.3.2. Behavioral Tests

##### Y-Maze Test

Blinding behavioral assessments is a crucial aspect of experimental design, so the behavioral tests were performed in a blinded manner, the rats being subjected to the same behavioral tests regardless of study group. The rats were trained on the behavioral tests for seven days before VRH administration. The animals were tested on the behavioral tests every day after LPS injection for seven days, and the different groups of rats were compared with each other.

The Y-maze test was conducted to evaluate the effects of the different treatments on the rats’ cognitive function. The Y-maze has three arms shaped like the letter “Y”. All the arms are identical in size and shape. During the test, the rat is placed at one branch of the maze and allowed to explore the environment over a period of 8 min. The sequence and frequency of entries into each arm were recorded. Total arm entries and spontaneous alternation were the two primary metrics that were measured. Rats’ inclination to investigate each arm in turn without repeating arm entries is known as spontaneous alternation. It is thought to be a reflection of working memory in space. Total arm entries show overall motor activity as well as exploratory behavior. The percentage of spontaneous alteration (SA%) was calculated by the following formula [83]:

number of spontaneous alteration performance (No. of SAP)/(Total arm entries − 2) × 100 (3)

##### Pole-Climbing Test

The pole-climbing test is used to evaluate brain-regulated locomotor function. In this experiment, the rat was placed with its head facing upwards at the top of a pole. The rat turned its head down in an attempt to descend. The time taken for the rat to turn (T_turn_) and how long it took to descend to the floor (TD) were recorded. In cases where the rats fell immediately, a maximum duration of 120 s was assigned to both Tturn and TD [84].

#### 4.3.3. Sample Collection

The rats were euthanized using light halothane anesthesia. The brain tissue was isolated, washed, divided into pieces, and stored at −80 °C for subsequent analyses. Some tissue samples were preserved in 7% formalin for histopathological examination and immunohistochemistry.

#### 4.3.4. Histopathological Examination

Fragments of the blocks were partitioned and stained with hematoxylin and eosin (H&E) to visualize the pathological distortions. The images were captured by a light microscope at ×400.

#### 4.3.5. Immunohistochemistry Examination

Following their embedding in paraffin blocks, the brain tissue samples were sectioned into thin slices, usually 4–6 μm thick. For immunostaining, the tissue sections were adhered to microscope slides. The sections were prepared for immunostaining by normal deparaffinization and rehydration procedures. TNF-α (1.5 µg/mL), IL-6 (2.5 µg/mL), nuclear factor kappa-light-chain-enhancer of activated B cells (NF-κB P65) (2.5 µg/mL), Toll-like receptor 4 (TLR4) (2.5 µg/mL), interferon gamma (IFN-γ) (1.5 µg/mL), and phosphorylated tau protein (pTau) (1.5 µg/mL) were all immunostained in the samples. To calculate the amounts of TNF-α, IL-6, NF-κB, TLR4, and pTau, average area percentages were used.

#### 4.3.6. Determination of Oxidative Stress Biomarkers

Glutathione (GSH), malondialdehyde (MDA), and nitrite were determined in the brain tissue by specific commercial colorimetric kits (cat. nos. E-BC-K030-S, E-BC-K025-S, and E-BC-K070-S, respectively) purchased from Elabscience^®^ (Houston, TX, USA). The GSH assay is based on the reaction of GSH with 5,5′-dithiobis (2-nitrobenzoic acid, DTNB), which produces a yellow-colored product that can be measured spectrophotometrically at 420 nm. The MDA assay is based on the reaction of MDA with thiobarbituric acid (TBA), which forms a pink-colored MDA-TBA adduct that can be detected spectrophotometrically at 532 nm. Nitrite is the main metabolic product of nitric oxide oxidation, and its determination depends on producing a red-colored compound detected at 550 nm.

Reactive oxygen species (ROS) were detected using a fluorometric assay kit (cat. no. E-BC-K138-F; Elabscience^®^, Houston, TX, USA) according to the manufacturer’s instructions. Detection depends on the presence of 2,7-dichlorofluorescin diacetate (DCFH-DA), which is a fluorescent probe without fluorescence that can readily diffuse through a cell membrane and hydrolyze and interact with intracellular ROS to give strong fluorescence signals. The signals were captured by an EVOS^™^ FLoid^™^ Cell Imaging Station (Thermo Fisher Scientific, Waltham, MA, USA), and fluorescence intensity was determined by Image J software Version 1.54 to estimate corrected total cell fluorescence (CTCF) as follows [83]:

integrated density − (area of selected cell × mean fluorescence of background readings) (4)

A heatmap was also constructed to reveal the range of CTCF for the studied groups.

#### 4.3.7. Determination of Amyloid Beta Protein

Different sequences of Aβ protein were determined in the tissue homogenates as specified in the manufacturer’s guidance. Commercial sandwich-ELISA kits specific for Aβ_1–40_ and Aβ_1–42_ were used (cat. nos. E-EL-R3030 and E-EL-R1402; Elabscience^®^, Houston, TX, USA) for the detection. A Multiskan SkyHigh Microplate Spectrophotometer (Thermo Fisher Scientific, Waltham, MA, USA) was used to measure the optical density at 450 nm.

#### 4.3.8. Determination of Gene Expression by qRT-PCR

The expression levels of CYP2B, cytochrome C oxidase (CCO), CREB-regulated transcriptional coactivator 3 (CRTC3), cAMP response element-binding protein (CREB), and brain-derived neurotrophic factor (BDNF) were determined by qRT-PCR. Entire RNAs were extracted from the tissue by the RNeasy^®^ mini kit (cat. no. 74104; Qiagen, Hilden, Germany) using QIAcube Connect (Qiagen, Hilden, Germany) according to the supplier’s protocol. Afterward, complementary DNAs were formed from the extracted RNAs by EasyScript^®^ First-Strand cDNA Synthesis SuperMix (cat. no. AE301-02; TransGen Biotech Co., Beijing, China). The reaction was amplified by a QuantiNova SYBR Green PCR Kit (cat. no. 208052; Qiagen, Hilden, Germany) as specified in the instructions using the StepOnePlusTM Real-Time PCR system (Thermo Fisher Scientific, Waltham, MA, USA). β-actin was used as a reference gene, the sequences of the primers were formed by Primer Blast, and they were bought from Macrogen (Seoul, Republic of Korea), as presented in Table S1. The results were estimated as relative quantifications (RQs).

#### 4.3.9. Determination of DNA Fragmentation by Comet Assay

A comet assay was performed on the brain tissues according to Singh et al. [85]. The tissue was chopped into tiny pieces in a chilled buffer containing 0.075 M NaCl and 0.024 M Na2EDTA, followed by homogenization. The suspension of the cells was centrifuged at 700× *g* for 10 min at 4 °C and re-suspended in a chill buffer, and the pellets were collected. Afterward, the cells were mixed with LMA and placed over frosted slides, which were placed in a lysis buffer for 1 h and then dipped in a neutralization buffer for 15 min. The samples were allowed to dry, stained with EP, and visualized by a microscope. Randomly selected fields of the slide (50–100 cells) were used to score the comet. DNA fragmentation was calculated as % DNA damage.

### 4.4. Statistical Analysis

Before conducting any analyses, the normality of continuous data was assessed using Kolmogorov–Smirnov and Shapiro–Wilk tests. Normally distributed data were presented as means ± standard deviations or standard error, while non-normally distributed data were expressed as medians (interquartile ranges). Parametric comparisons among the groups were performed using one-way analysis of variance (ANOVA) followed by Tukey’s post hoc test. Non-parametric data were analyzed using the Kruskal–Wallis test, with Dunn’s test used for post hoc correction (SPSS version 22). The results were considered statistically significant when *p* < 0.05. Figures were constructed by GraphPad Prism software version 10.1.0.

## 5. Conclusions

Verapamil showed multifaceted mechanisms in combating LPS-induced neurotoxicity through its anti-inflammatory and antioxidant properties, thereby mitigating the hallmarks of AD, Aβ, and pTau deposition, with amelioration of cognitive dysfunction. Moreover, our synthesized nanosystem approach exhibited superior neuroprotective effects compared to the free VRH owing to the advantages offered by CQDs. The early intervention revealed better restoration of the deteriorated biomarkers, which proved the implication of inflammation, oxidative stress, and the CRTC3/CREB/BDNF signaling pathway in the development of AD. However, finding new actionable biomarkers and molecular targets is of decisive importance to improve the outcomes for patients with AD.

## Figures and Tables

**Table 1:** Physicochemical characterization of the prepared verapamil-loaded carbon quantum dots.

Formula	Quantum Yield (%) ^a,e^	Particle Size (nm) ^b,e^	PDI ^b,e^	Zeta Potential (mV) ^c,e^	AE% ^d,e^
VCQDs	15.65 ± 2.54	7.5 ± 0.54	0.18 ± 0.012	−16.5 ± 1.35	81.25 ± 3.65

^a^ Quantum yield % was measured by a spectrofluorometer using quinine sulfate as a reference. ^b^ Particle sizes and polydispersity indices (PDIs) were measured using the dynamic light scattering technique. ^c^ Zeta potential was measured using electrophoresis. ^d^ The association efficiency percentage was calculated as a percentage of the initial VRH added, determined directly by HPLC. ^e^ Expressed as means ± SDs (n = 3).

**Table 2:** Histological changes in the pretreated groups.

	Group	LPS	CQDs	Early FVRH	Late FVRH	Early VCQDs	Late VCQDs
Lesions							
Polymorphic layer degeneration		5	5	3	3	1	2
Pyramidal cell layer degeneration		7	8	4	5	2	3
Septum lucidum layer degeneration		8	7	3	3	1	2
Molecular layer degeneration		7	6	3	4	1	2
Vacuolation		8	7	5	4	1	3
Blood capillary degeneration		7	7	3	4	1	2
Perineural dark oligodendrocytes		7	8	2	3	0	0
Large light-stained nuclei		5	4	1	1	0	0
Apoptotic cells		5	6	2	3	1	1
Pyknotic nuclei		8	7	5	4	2	1
Karyorrhexic nuclei		7	8	1	2	0	0

Data are presented as means ± SDs, n = 6 per group; no (0), slight (1), rare (2), low (3), mild (4), moderate (5), high (6), very high or strong (7), and very strong changes (8). All the treated groups received LPS. LPS: lipopolysaccharide, CQDs: hyaluronic acid caron quantum dots, FVRH: free verapamil, VCQDs: verapamil-loaded CQDs.

## Data Availability

Data are available upon reasonable request.

## Supplementary Materials

The following supporting information can be downloaded at: https://www.mdpi.com/article/10.3390/ijms25147790/s1.
