# Supplementary material for: Mechanistic Insights of Neuroprotective Efficacy of Verapamil-Loaded Carbon Quantum Dots against LPS-Induced Neurotoxicity in Rats
Source: Int J Mol Sci. 2024 Jul 16;25(14):7790. doi: 10.3390/ijms25147790 (PMC11277230; doi:10.3390/ijms25147790)
Supplement: Supplementary file 1 [file ijms-25-07790-s001.zip › ijms-3083688-supplementary.pdf]

Table (S1): Sequence of the used primers

Gene	Forward	Reverse
**CYP2B**	AGGAGTGTGGTGTGGGCAAA	CTACAGAGGCGGGGACACTG
**CCO**	CTCCAAGCGCCCGAGAAGTA	CCATGGCGGAAGTGGGAGAT
**CRTC3**	CTGAGCACCAAACCCCAGGA	GGGGAAGGCGGCCATGTAT
**CREB**	CCAGGGGTGCCAAGGATTGA	GCTCCTCCCTGGGTAATGGC
**BDNF**	CCCCGCACACTCTGTGTAGT	GCAGCCTTCATGCAACCGAA
**β-actin**	CCCAGCCATGTACGTAGCCA	CCGTCTCCGGAGTCCATCAC

CYP2B: cytochrome P450 2B, CCO: cytochrome c oxidase, CRTC3: CREB-regulated transcriptional coactivator 3, CREB: cAMP response element-binding protein, BDNF: and brain derived neurotrophic factor.

Table (S2): Median and interquartile range of the detected genes in the studied groups

Parameter / Group	Gene	Median	Interquartile range
**Normal control**	CYP1A1	0.8984	0.98
	CCO	1.1549	0.55
	CRTC3	1.1208	1.35
	CREB	1.5540	1.15
	BDNF	1.1208	1.35
**LPS**	CYP1A1	0.0001 [a]	0.00
	CCO	0.0002 [a]	0.00
	CRTC3	0.0014 [a]	0.01
	CREB	0.0010 [a]	0.00
	BDNF	0.0014 [a]	0.01
**CQDs**	CYP1A1	0.0007 [a]	0.00
	CCO	0.0013 [a]	0.00
	CRTC3	0.0058 [a]	0.01
	CREB	0.0031 [a]	0.01
	BDNF	0.0058 [a]	0.01
**Early free VRH**	CYP1A1	0.3234 [b, c]	0.36
	CCO	0.0063 [a]	0.01
	CRTC3	0.1070 [b, c]	0.17
	CREB	0.0801 [a, b]	0.11
	BDNF	0.1070 [a, b, c]	0.17
**Late free VRH**	CYP1A1	0.0229 [a]	0.04
	CCO	0.0020 [a]	0.01
	CRTC3	0.0389 [a]	0.08
	CREB	0.0657 [a, b]	0.10
	BDNF	0.0389 [a]	0.08
**Early VCQDs**	CYP1A1	0.8017 [b, c, e]	0.92
	CCO	0.5822 [b, c, d, e]	1.80
	CRTC3	0.2640 [b, c, e]	0.50
	CREB	0.7649 [b, c, d, e]	1.24
	BDNF	0.2640 [b, c, e]	0.50
**Late VCQDs**	CYP1A1	0.1949 [b, c]	0.39
	CCO	0.0636 [b, c, e]	0.10
	CRTC3	0.1667 [b, c]	0.24
	CREB	0.2831 [b, c]	0.64
	BDNF	0.1667 [b, c]	0.24

Kruskal-Wallis test followed by Dunn's test between groups. n = 6, $P < 0.05$. a: significant *vs.* normal control, b: significant *vs.* LPS, c: significant *vs.* CQDs, d: significant *vs.* early FVRP, e: significant *vs.* late FVRP, f: significant *vs.* early VCQDs. LPS: lipopolysaccharides, CQDs: hyaluronic acid caron quantum dots, FVRH: free verapamil, VCQDs: verapamil-loaded CQDs, CYP1A1: cytochrome P1A1, CCO: cytochrome c oxidase, CRTC3: CREB-regulated transcriptional coactivator 3, CREB: cAMP response element-binding protein, BDNF: brain-derived neurotrophic factor.
